# Seasonal Influenza Vaccination amongst Medical Students: A Social Network Analysis Based on a Cross-Sectional Study

**DOI:** 10.1371/journal.pone.0140085

**Published:** 2015-10-09

**Authors:** Rhiannon Edge, Joseph Heath, Barry Rowlingson, Thomas J. Keegan, Rachel Isba

**Affiliations:** Department of Health and Medicine, Lancaster University, Lancaster, Lancashire, United Kingdom; University of Bristol, UNITED KINGDOM

## Abstract

**Introduction:**

The Chief Medical Officer for England recommends that healthcare workers have a seasonal influenza vaccination in an attempt to protect both patients and NHS staff. Despite this, many healthcare workers do not have a seasonal influenza vaccination. Social network analysis is a well-established research approach that looks at individuals in the context of their social connections. We examine the effects of social networks on influenza vaccination decision and disease dynamics.

**Methods:**

We used a social network analysis approach to look at vaccination distribution within the network of the Lancaster Medical School students and combined these data with the students’ beliefs about vaccination behaviours. We then developed a model which simulated influenza outbreaks to study the effects of preferentially vaccinating individuals within this network.

**Results:**

Of the 253 eligible students, 217 (86%) provided relational data, and 65% of responders had received a seasonal influenza vaccination. Students who were vaccinated were more likely to think other medical students were vaccinated. However, there was no clustering of vaccinated individuals within the medical student social network. The influenza simulation model demonstrated that vaccination of well-connected individuals may have a disproportional effect on disease dynamics.

**Conclusions:**

This medical student population exhibited vaccination coverage levels similar to those seen in other healthcare groups but below recommendations. However, in this population, a lack of vaccination clustering might provide natural protection from influenza outbreaks. An individual student’s perception of the vaccination coverage amongst their peers appears to correlate with their own decision to vaccinate, but the directionality of this relationship is not clear. When looking at the spread of disease within a population it is important to include social structures alongside vaccination data. Social networks influence disease epidemiology and vaccination campaigns designed with information from social networks could be a future target for policy makers.

## Introduction

Globally, influenza affects an estimated 5 million people per year[1]. Influenza causes considerable morbidity each year and can be a primary, underlying, or contributing factor to cause of death[2]. Seasonal influenza vaccination remains the best method of preventing influenza spread through populations by generating herd immunity[3]. Many researchers in this area suggest that higher vaccination coverage within a hospital will have positive benefits such as: a reduction in patient mortality, less staff absence, and a reduction in influenza transmission within long-term care homes [4–8]. Despite this, in 2013, only 55% of healthcare workers (HCWs) had a seasonal influenza vaccine, and uptake varied across different healthcare trusts in England and Wales. This figure was also considerably lower than the 75% target set by the Chief Medical Officer (CMO) for England [9]. Commonly cited reasons for HCWs not having seasonal influenza vaccination include: a lack of knowledge about the vaccine, fear of side effects, doubts about risk of influenza infection, concerns about vaccine effectiveness, and a dislike of injections [10–12]. Educational campaigns designed to address these issues appear to have had little effect on uptake amongst HCWs [13]. There is a need, therefore, for novel approaches to improving seasonal influenza vaccination uptake amongst this group. Social network analysis approaches have been used to study the uptake of other vaccinations in different populations, so lend themselves to the study of seasonal influenza vaccination.

Social network analysis (SNA) stems from the concept that an individual’s actions are affected by their peers and that people should therefore be studied in their wider social context. A network is formed from individuals (nodes) connected via ties (relationships). Social network models are increasingly being applied to epidemiological studies of, for example, effects on smoking cessation, and in the investigation of sexually transmitted diseases[14, 15]. Using a SNA approach to study how social structure relates to vaccination attitudes, vaccine uptake, and the effects on influenza transmission among medical students is a novel approach.

In human populations, individuals rarely mix randomly—there is often influence (positive or negative) from social structures. For example, recent work has shown that the percentage of contacts in a parent’s social network recommending non-conformity has been found to be a negative predictor for childhood vaccination[16]. In a work setting, HCWs seem to be positively influenced by their co-workers’ vaccination practices[17]. This phenomenon of imitation, has been observed elsewhere in relation to vaccination—a recent study of US high school students found that individuals who vaccinated were more likely to form connections with others who also vaccinated [16, 18]. Non-random distribution of vaccination in a population (vaccination homophily) is thought to increase the likelihood of infectious disease spread within that population, as clusters of non-vaccinated individuals can form reservoirs from which disease may spread[19–21]. Increasingly, researchers in infectious disease epidemiology are recognising the importance of including a contact network when modelling influenza outbreaks [22].

At Lancaster Medical School (LMS) students are encouraged to have a seasonal influenza vaccination in a way that mirrors the recommendations given to HCWs. We used a SNA approach to look at vaccination distribution within the network of the LMS student population and combined these data with the students’ beliefs about the vaccination behaviours of others. We then developed a model which simulated influenza outbreaks in the population to study the effects of preferentially vaccinating well-connected individuals within this network.

## Methods

### Ethics, consent and permissions

Prospective ethical approval was obtained from Lancaster University Research Ethics Committee and, following a verbal and written explanation of the study, each participant gave informed consent prior to taking part. The study was performed in accordance with the Declaration of Helsinki. Identifiable data were collected using a standard SNA approach and then anonymised prior to entry and analysis.

### Study population

Lancaster Medical School (LMS) is in the North West of England and offers a primary undergraduate medical degree course over five years of study. At the start of the academic year 2013/14, there were 253 students actively enrolled in the course. All students were invited to participate in the data collection which took place over two months at the end of term 1 and the start of term 2.

### Data collection

Each participant gave written consent and completed a paper-based data collection instrument. In addition to providing basic demographic information (age, sex, and year of study), participants were also asked to indicate whether they had received the seasonal influenza vaccination. They were also asked what percentage of medical students they thought had also received the vaccine. A copy of the questionnaire is available in the online appendix.

Individuals were also asked to rate the strength of their relationship with every other student in the medical school on a six-point scale:

- 0—“I do not recognise this name”
- 1—“I recognise this name but I would not recognise the person if I saw them”
- 2—“I would recognise the person if I saw them”
- 3—“I know this person and see or speak to them once or twice a week”
- 4—“I know this person and see or speak to them three or more times a week”
- 5—“I live with this person/have a close relationship with them/socialise with them on a regular basis”

This scale was used to gain an understanding of how much time students spent with others in the medical school without having to rely too heavily on participant recall (participants were provided with the names of everyone enrolled in the school). We wanted to include the strongest ties for analysis, however, when the data was dichotomised at level 5 isolates appeared (these individuals would have then been removed from much of the analysis), so we dichotomised the data at level 4 and above.

### Missing Data

We dealt with missing data by assigning the reciprocal score where a pair of individuals had rated each other only once i.e. if person *x* had not completed the data collection instrument and person *y* had rated their relationship with a strength of 5, we assumed that *x* would also rate person *y* with a 5. Where neither party had provided data relating to the relationship, an assumption was made that there was no relationship. Both of these approaches are considered acceptable when constructing a social network with a high response rate[23]. For the influenza vaccination data, we used three possible states of vaccination for the network—vaccinated, unvaccinated, and unknown (no response)–but for the outbreak model, these options were reduced to vaccinated and unvaccinated (which included those who had not provided a response).

### Social Network Analysis

Data were inputted into a Microsoft Excel file after being anonymised. The relationship data were then converted to numerical data, using the values outlined above, and inputted into an adjacency matrix. The adjacency matrix for the medical school had 253 rows and 253 columns—corresponding to the number of students in the school. The social network of the medical students was then dichotomised so that it only contained ‘close’ relationships i.e. students who met frequently and were therefore more likely to influence each other. Thus, only relationships categorised as: “I know this person and see or speak to them three or more times a week” or “I live with this person/have a close relationship with them/socialise with them on a regular basis” were included in this analysis. These were thought to be the most likely contacts to facilitate infectious disease spread, and we assume that these contacts would be more likely to influence the individual compared with others in the LMS network that they did not meet frequently. The final adjacency matrix therefore contained only 0s and 1s. Reciprocal ties were assumed, according to standard practice in this type of social network analysis so regardless of what the other party had answered if one person in the pair had rated the relationship as a 4 or a 5, a tie was constructed [24]. All subsequent statistical analysis and network visualisation was carried using R statistical software with igraph [25].

The adjacency matrix was then used to create and visualise a simple (nodes cannot form ties with themselves), undirected (all ties were assumed reciprocal), un-weighted (all ties post-dichotomisation were either present or absent) network. Vaccination data were then mapped onto the social network data. Data relating to students’ beliefs about the vaccination status of others were handled separately.

To investigate whether clusters of vaccinated or unvaccinated individuals existed we measured the assortative mixing of participants based on their vaccination decision, by calculating the assortativity coefficient. Assortativity is a standard network measure developed by Newman[26] and calculation of this measure produces a value between -1 and 1, with values tending to -1 indicating negative assortativity, 0 indicating random (or no) assortativity, and 1 indicating positive assortativity within the network (see technical appendix). Assortativity was calculated for the entire medical student population as well as for individual year groups. Additionally, a sensitivity analysis was carried out to look at the effect on assortativity of dichotomising the data at a value of greater than equal to 3 and 5, as well as the main study threshold of 4.

Each individual’s influence on the network was measured in terms of how well connected they were within the network. SNA defines multiple measures for connectivity—we scored individuals based on their degree and between-ness. In a network, an individual’s degree is simply the number of connections they possess [27] and the between-ness score is based on the extent to which they are able to act as a ‘gate-keeper’ between the others in the network. A high between-ness score indicates that an individual lies between groups and is therefore important for the transmission of information or disease between the groups. Between-ness is calculated for each individual as the number of most efficient paths from every student to every other student, that pass through the individual [27].

### The individual-based outbreak simulation model

An individual-based outbreak model was developed using R. Using the network data we simulated an influenza outbreak and assessed the effects of preferentially vaccinating according to the social network analysis data. The probability that an individual was infected at any given time step was dependent on: the adjacency matrix, their vaccination status, and appropriate transmission parameters derived from the literature—these were based on a discrete time step of one day.

Transmission parameters were based on the following assumptions:

- relationships—for the purposes of the simulation, students were judged as having a ‘close’ relationship if they lived together or met more than three times a week (see above)
- the literature suggests that 11% of un-vaccinated close contacts of cases become infected with the influenza virus based on the H1N1 equivalent (probability 0.11)[28]
- the transmission probability was reduced by 74% (probability 0.0286) for students who chose to vaccinate, based on previous reported vaccine efficacy[29]
- recovery—the probability of recovery was set at 0.334, based on a recovery time of 3 days and did not vary within the population. If an infected individual moved into the recovered stage, they could no longer infect others or themselves be infected a second time.

We identified un-vaccinated individuals with the highest scores for between-ness and degree and additionally vaccinated them to test the effects on the simulated outbreak of influenza. We also ran the simulation with randomly selected un-vaccinated people to vaccinate, as a control. During each run of the simulation, the outbreak was allowed to continue until it became unsustainable i.e. burned out on its own. During each trial the simulation ran 1500 times, with each simulation selecting a random student to introduce the virus into the population. At every time step the infected individuals were given the opportunity to transmit the infection to their susceptible neighbours (the probabilities of infection are given above). For each vaccination strategy, the likelihood of each individual in LMS being infected was calculated as a percentage of the number of times they were infected in the 1500 possible outbreaks. The Kolmogorov-Smirnov test was used to show whether there was any significant difference in the likelihood that individuals in the LMS would contract infection when students were vaccinated according to either their between-ness or degree scores, or randomly.

## Results

### Initial data exploration

**Fig 1:**
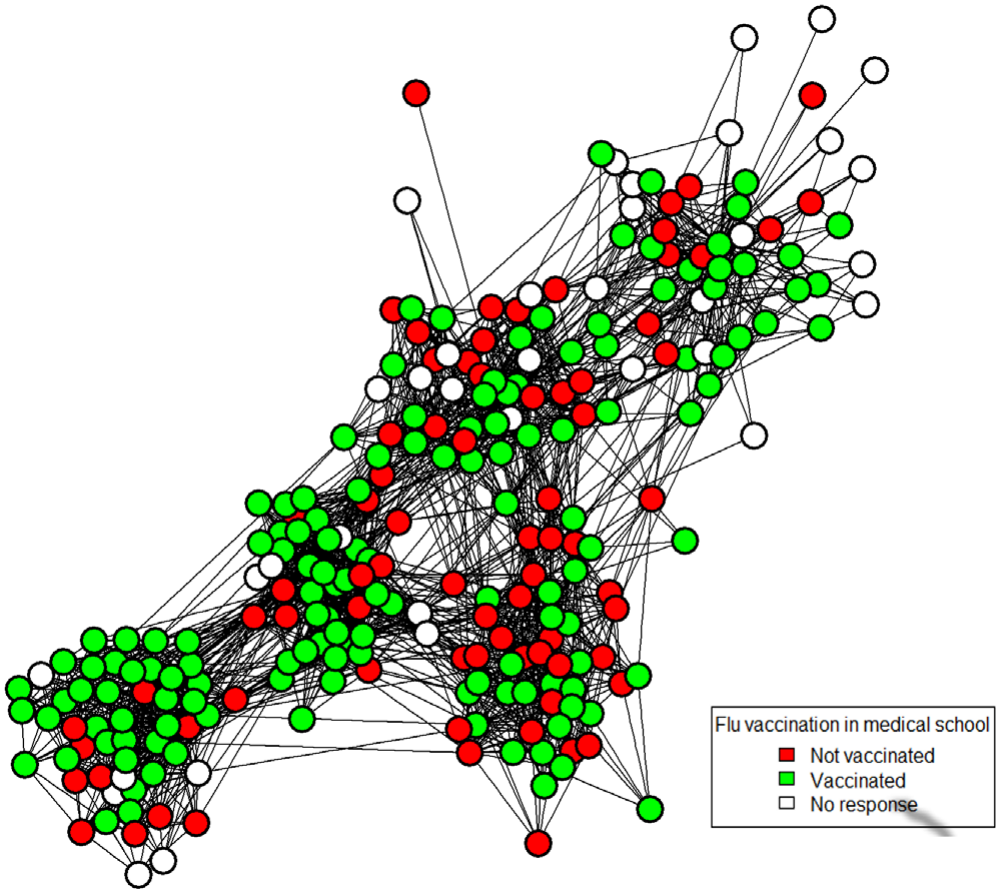
Network map of Lancaster Medical School. A network map of the Lancaster Medical School social network dichotomised at 4 (“I know this person and see or speak to them three or more times a week”) and above. Seasonal influenza vaccination status is superimposed: non-vaccinated are red; vaccinated are green; and non-responders are white.

Of those studying at LMS (n = 253), 217 (85%) participated in this study. Non-participants were mainly in the final two years of study (response rates: Year 1 89%, Year 2 89%, Year 3 100%, Year 4 80%, and Year 5 70%). Participants were aged between 18 and 38. Sixty-five percent of respondents (141/217) had received a seasonal influenza vaccination, which represented 56% (141/253) of the entire network (76/253 unvaccinated and 36/253 unknown). Fig 1 shows the entire medical student social network, dichotomised at a value of 4 (corresponding to the statement “I know this person and see or speak to them three or more times a week”), with the vaccination data superimposed on the structure.

### Measures of connectivity—between-ness and degree

**Fig 2:**
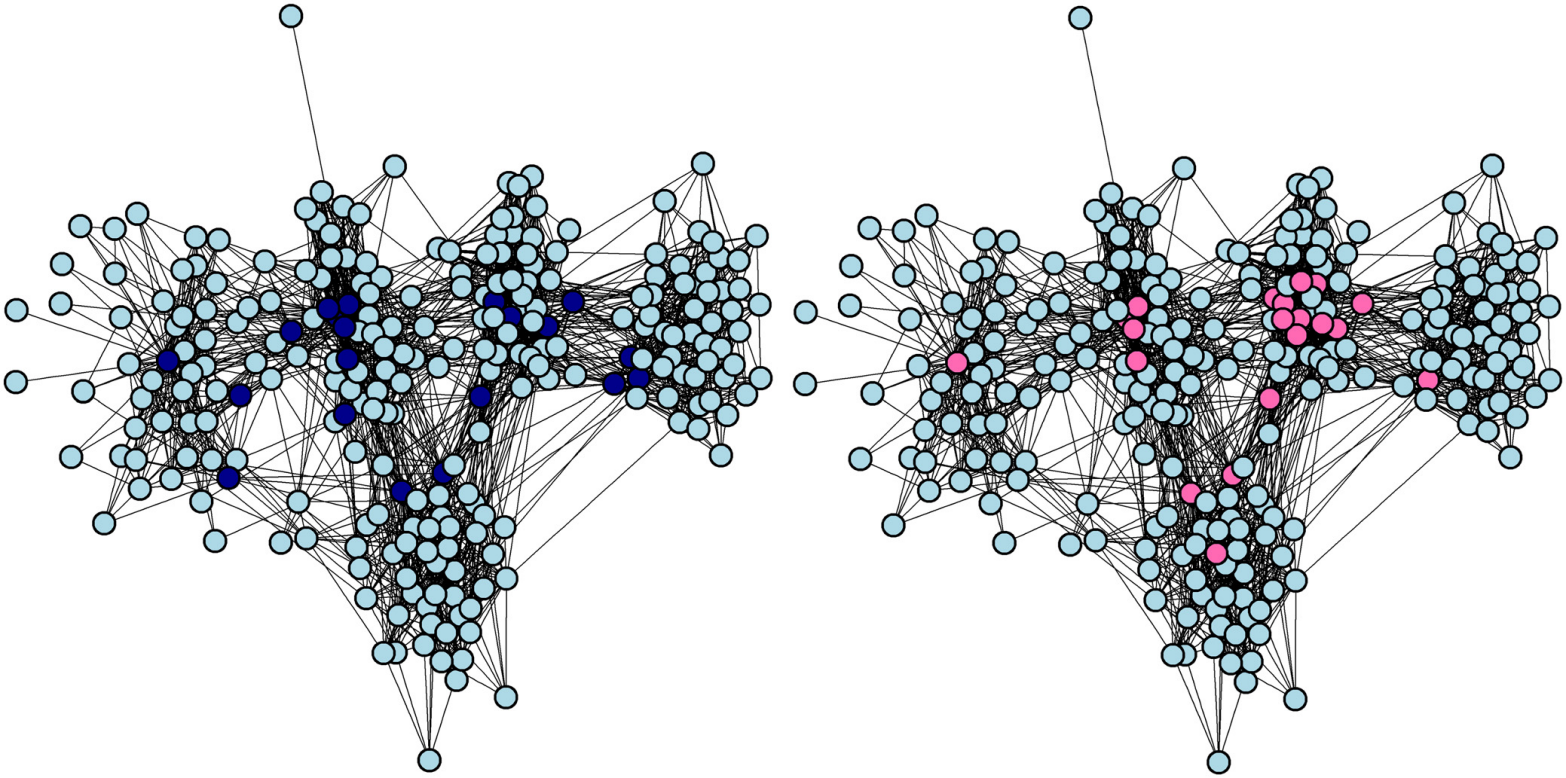
Well-connected individuals in the medical school. The LMS social network, dichotomised at 4 (“I know this person and see or speak to them three or more times a week”) and above. On the left, individuals with the 20 highest scores for between-ness are highlighted in dark blue, and on the right, individuals with the 20 highest degree scores are highlighted in pink.

When dichotomised at 4 and above, the mean degree (number of contacts per person) was 18 (range 1 to 57) and year groups tended to cluster together. Fig 2 highlights the individuals who had the top 20 scores for between-ness and degree. Both these measures assess connectivity in a different way. This can lead to different sets of people being identified as the most connected; in this case there was some overlap between the groups.

**Fig 3:**
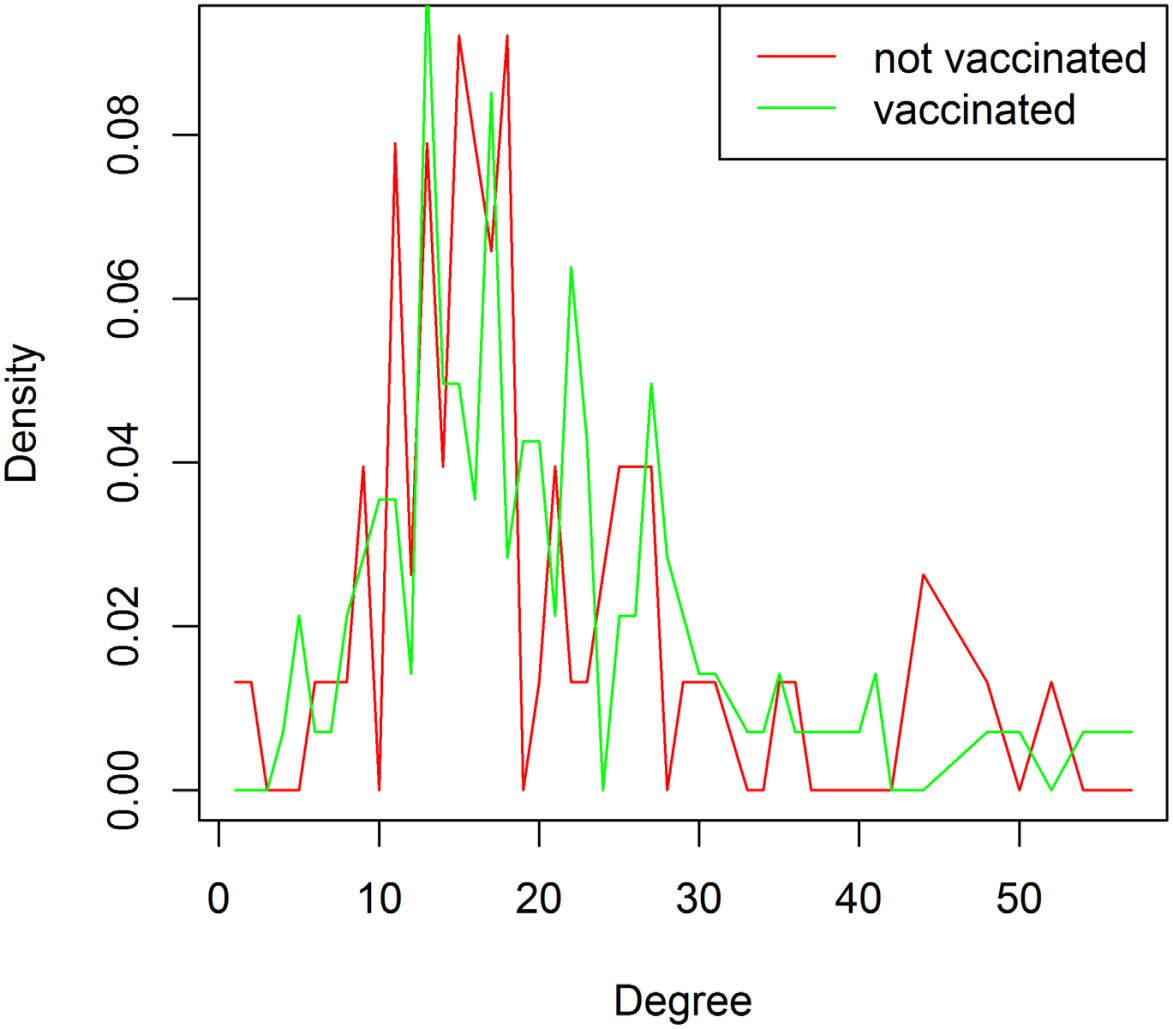
Degree density plot. The degree density of vaccinated (green) and non-vaccinated (red) individuals appears similar.

We compared vaccination between the most well-connected individuals (i.e. those with a high degree) and the least well-connected individuals (i.e. those with a low degree). Thirteen of the 20 (65%) most well-connected individuals were vaccinated and 12 of the 20 (60%) least well-connected individuals were vaccinated. Fig 3 shows that the degree density plots for vaccinated and non-vaccinated individuals appear very similar. Applying the Kolmogorov-Smirnov test to these data yielded a non-significant p-value (D = 0.24444, p-value = 0.1359) thus we cannot reject the hypothesis that these two distributions are similar.

### Perception of Influenza vaccination coverage

**Fig 4:**
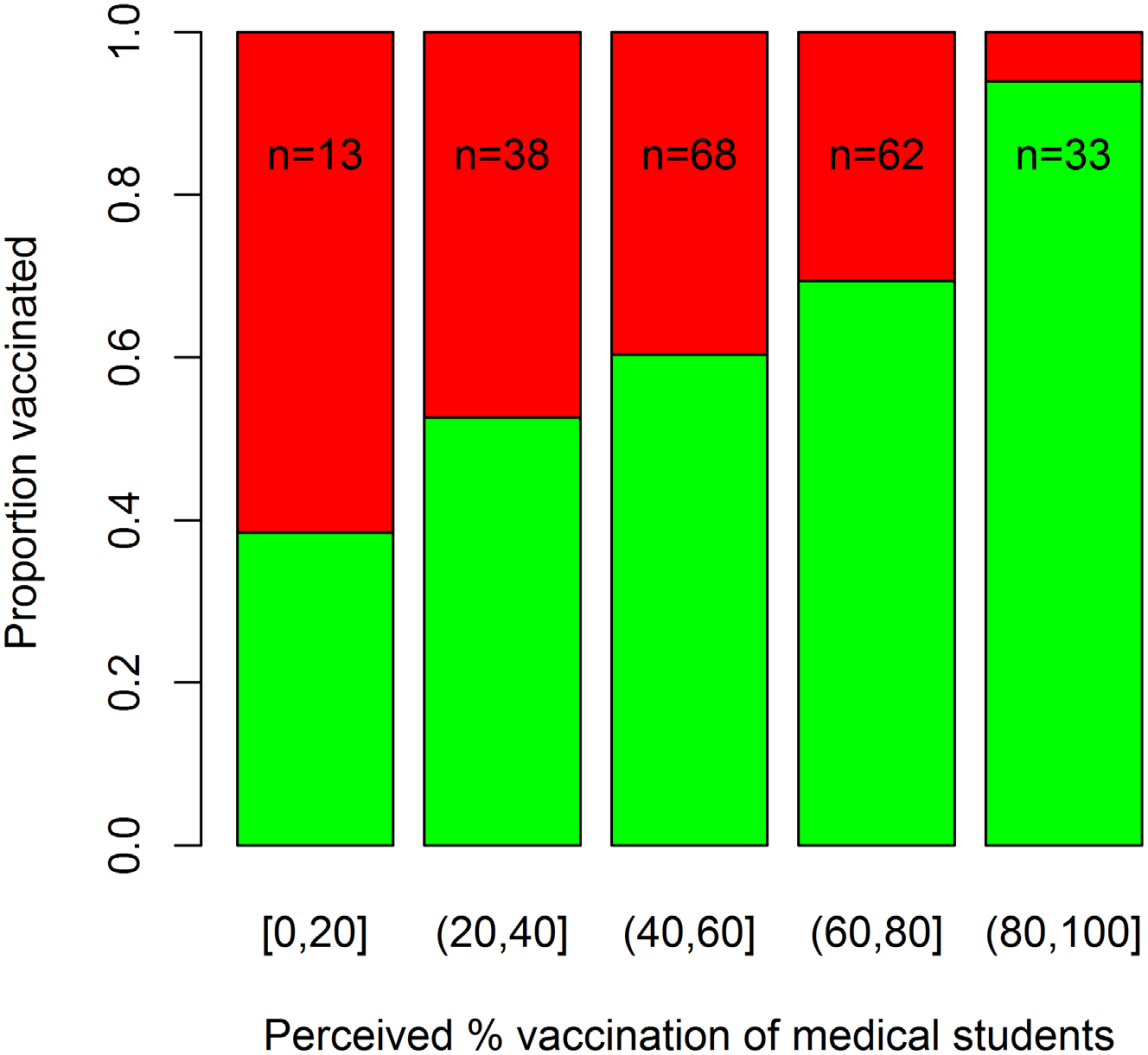
Perception of vaccination within the medical school. Graph showing perception of vaccination and the vaccination coverage within each group. Green represents the vaccinated individuals within each group and red the non-vaccinated. The groups are divided up by level of perceived vaccination coverage and the number of students that fall into each group is given at the top of each bar.

Fig 4 shows the medical students’ perceptions of their peers’ vaccination behaviours. On average, participants believed that 60% of other students at LMS were vaccinated (range 10–100%). When students were grouped based on their perceptions of the vaccination status of others, we found that an individual’s vaccination status correlated with perception—i.e. students suggesting high levels of vaccination amongst their peers were themselves more likely to be vaccinated (and the reverse was also true, Fig 4).

### Assortativity

Assortativity of vaccination status was used to test whether individuals preferentially connected with others with similar vaccination status, and to identify any clusters or pockets of highly vaccinated or un-vaccinated students. In this study, assortativity coefficients for vaccination were consistently around zero, regardless of year group or level of dichotomisation, indicating near-random mixing between vaccinated and un-vaccinated students (Table 1).

**Table 1:** Vaccination assortativity at Lancaster Medical School, stratified at various levels of contact.

	Year group	1	2	3	4	5	All years
Contact level	5	0.02	-0.10	-0.03	-0.01	-0.09	0.00
	4 and above	0.01	0.02	0.01	-0.06	-0.06	0.03
	3 and above	-0.03	-0.01	-0.02	-0.07	-0.07	0.01

### Outbreak simulation results

**Fig 5:**
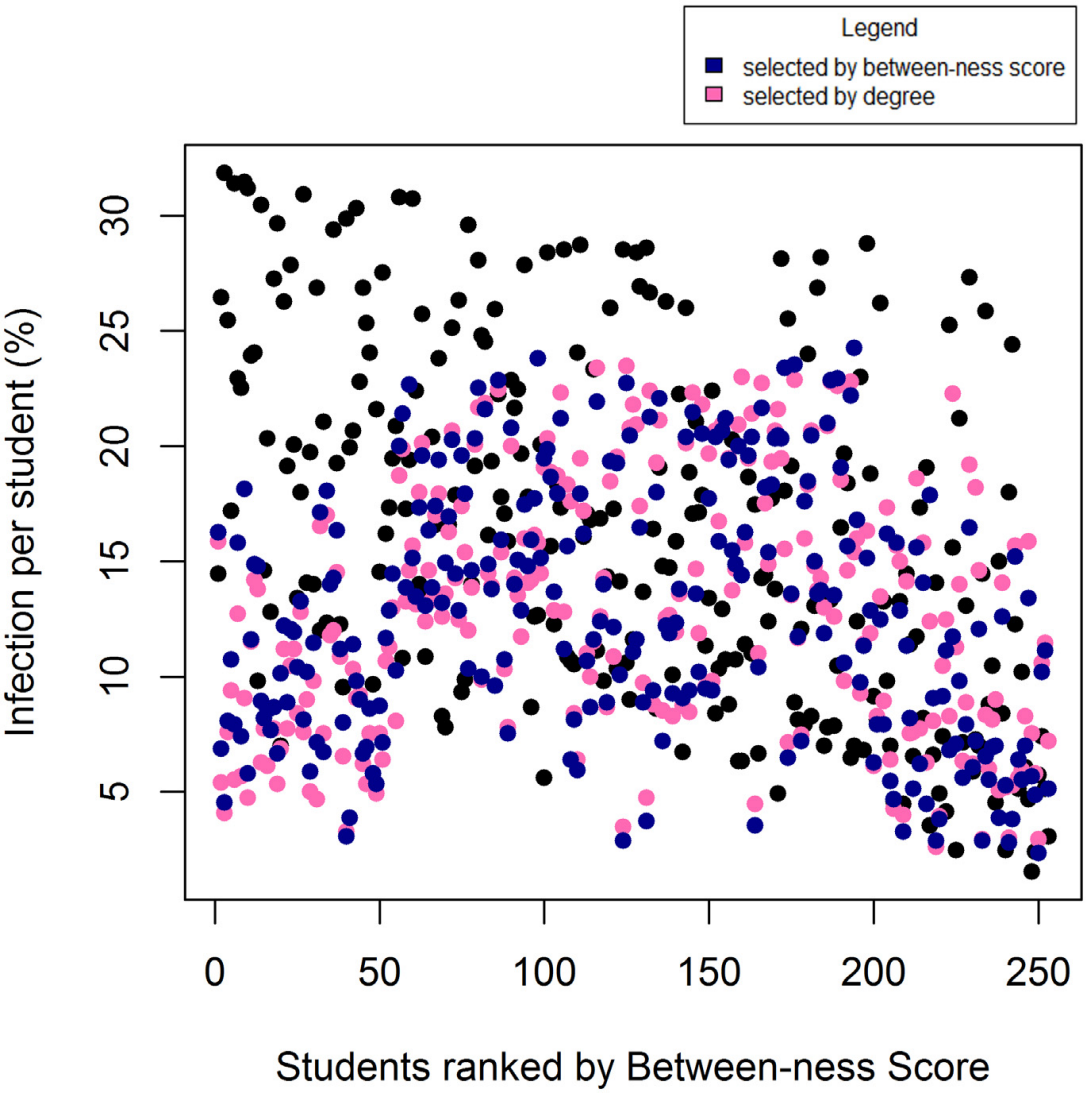
Influenza outbreak simulation results. A scatter plot showing the percentage number of times each individual was infected during the 1500 influenza outbreak simulations. During the conditions: random vaccination (black); vaccination by between-ness (blue); and, vaccination by degree (pink).

From the outbreak model we found that the effect of vaccinating additional individuals based on between-ness and degree were similar i.e. the likelihood of individuals contracting the infection tended to be similar, irrespective of vaccination based on between-ness or degree. As an example, Fig 5 shows the results of the outbreak simulation following the vaccination of an additional 20 students according to either their between-ness score, their degree, or randomly. After vaccination of this additional 8% of the population (20 students), the outcome of the experimental influenza outbreak was similar for between-ness and degree, and considerably better than a random vaccination policy. The Kolmogorov-Smirnov test confirmed this, when the results from the simulation under random vaccination and vaccination according to between-ness were compared, we rejected the hypothesis that the two distributions were similar (D = 0.22925, p-value < 0.0001). When comparing the results under the random and between-ness vaccination policies we could not reject the hypothesis that the two distributions were similar (D = 0.035573, p-value = 0.9972).

## Discussion

Vaccine uptake among participants was 65%—slightly higher than in the wider medical community [9]. In contrast to work carried out in other populations, we found that this medical student population did not display vaccination assortativity—individuals at LMS who were vaccinated were just as likely to befriend non-vaccinated individuals as vaccinated individuals. We found a correlation between a student’s perception of the vaccination coverage amongst their peers and their own decision to vaccinate. The influenza simulation model demonstrated that well-connected individuals potentially have a disproportional effect on disease dynamics and may provide a focus for targeted intervention.

This study represents a novel approach to looking at seasonal influenza vaccination uptake and outlines potential benefits of this as a methodological approach in infectious disease epidemiological. Whilst missing data is a common limitation in social network studies we dealt with this by using a robust approach that, combined with a high response rate, means that we can be confident in our findings. The simulation model presented in this study is a method of testing the effects of vaccination policy changes that would otherwise be almost impossible to implicate and measure in the real world. The major limitation of this study is that we have artificially created a closed network made up of the LMS medical student body and assumed that meaningful contact only takes place within this network. In reality, medical students interact on a daily basis with a wide variety of individuals outside of the student body, and these interactions will be especially important in the context of disease transmission. Additionally, students are likely to be influenced by a variety of other people, and these influences may have a direct (or indirect) impact on decisions they make around vaccination. This study used social network data as a proxy for the contact network for the purposes of the simulation model. Adequate data collection for the true contact network is an ongoing problem in this field and requires further research. Alternative approaches to data collection include the use of wireless sensors that detect face-to-face contact between participants. These avoid reporting bias and some measurement error. However, this method also has limitations:, the network is limited to participants only, and it is generally feasible only to record the network for a limited time period.. The data collection method implemented here is a cheaper and simpler alternative, suitable to this study. However, more research is needed to establish the most effective data collection tools. Other limitations of this study are the choice and number of assumptions made when setting transmission parameters—for example, no attempt was made to include differing contact between days and times of day (both of which have an impact on disease transmission)–and the fact that all the data presented are self-reported.

The LMS medical student population did not display vaccination assortativity and this finding is at odds with previous similar studies in other populations[18]. There are many possible explanations for this observation, for example, it may be the case that the social dynamics within the medical student network are sufficiently different from other networks as to produce different vaccination structures. Equally it may be that the nature of seasonal influenza vaccination is such that decision-making relating to it is different compared to routine childhood vaccination for example. Only the population of interest was studied, individual’s ego-centric data was not collected. The external factors influencing medical students’ decisions around seasonal influenza vaccination may have had an impact on assortativity that has not been examined here. Extending this investigation to include ego-centric data is a natural progression of this study and may lead to an explanation for how vaccination assortativity develops in a population and why we might find differences in assortativity. Previous research has suggested that populations with strong vaccination assortativity can lead to an increased vulnerability to disease due to the formation of reservoirs of unvaccinated individuals. Therefore, the lack of assortativity seen at LMS may actually lower the risk of an influenza outbreak compared to other populations. More research is needed to determine the drivers of vaccination assortativity and to examine it in a wider context.

An individual student’s perception of the vaccination coverage amongst their peers appears to correlate with their own decision to vaccinate, but the directionality of this relationship is not clear. It may be that either vaccinated individuals perceive higher vaccination coverage amongst their peers or that people who perceive a higher vaccination in their peers get vaccinated as a form of imitation behaviour. It is worth noting that students did not appear to have very good knowledge of true vaccination uptake generally and this has implications for those seeking to increase seasonal vaccination uptake rates in other healthcare professionals. We speculate that it may be possible to increase vaccination uptake by educating individuals about vaccination coverage—perhaps encouraging individuals to vaccinate because their co-workers have. However, game theory might suggest the opposite—if an individual thinks that a large proportion of their immediate neighbours are vaccination, they may choose not to vaccinate instead relying on herd immunity[30]. Understanding the effects of peer influence could have huge implications for a range of public health policies. Our results suggest that there is little difference in the degree distribution between vaccinated and non-vaccinated individuals. When looking at the spread of disease within a population it is important to include social structures alongside vaccination data and this work supports the argument for the inclusion of social network analysis in epidemiological studies of infectious disease outbreaks. The simulation model we have developed shows that outcomes of vaccination strategies based on selecting individuals based on between-ness and degree were similar, and both were better than a random vaccination policy. This is important as vaccination campaigns targeting individuals based on a high degree score would be preferential due to its relative ease of calculation. Further investigation into well-connected individuals is necessary—profiling these individuals may highlight demographic proxies for influence—campaigns could then be targeted based on these.

## Conclusions

Considering past work in this area and the results outlined here it is clear that social networks influence disease epidemiology—thus vaccination campaigns designed with information from SNA provide a potential target for policy makers. Future work should include further empirical analysis of HCWs, working towards a better understanding of social network phenomena such as assortative mixing or influential individuals.

## Supporting Information

S1 File Technical Appendix. (DOCX)

S2 File Social network data. (CSV)

S3 File Influenza vaccination data. (CSV)

S4 File Questionnaire. (PDF)

## Abbreviations

SNA: Social Network Analysis
CMO: Chief Medical Officer
HCW: Healthcare Worker
LMS: Lancaster Medical School
