# Supplementary material for: Seasonal Influenza Vaccination amongst Medical Students: A Social Network Analysis Based on a Cross-Sectional Study
Source: PLoS One. 2015 Oct 9;10(10):e0140085. doi: 10.1371/journal.pone.0140085 (PMC4599893; doi:10.1371/journal.pone.0140085)
Supplement: S1 File — (DOCX) [file pone.0140085.s001.docx]

**Technical appendix**

- 1. **Assortativity calculation**

Assortativity is a standard network measure developed by Newman [1];

$$r= \frac{\sum_{i} e_{ii}- \sum_{i} a_{i}b_{i}}{1- \sum_{i} a_{i}b_{i}}$$

$where, e_{ij} is the fraction of edges connecting vertices of type i and j, a_{i}= \sum_{j} e_{ij} and b_{j}= \sum_{i} e_{ij},$

the value obtained can lie anywhere between -1 and 1, with values tending to -1 indicating negative assortativity, 0 indicating random assortativity and 1 indicating positive assortativity within the network .

- 1. **Between-ness formula**

A node’s between-ness score is based on the extent to which they are able to act as a ‘gate-keeper’ between the others in the network, and is calculated using:

$$\left( P_{k} \right)=\sum_{i\neq P_{k}\neq j} \frac{g_{ij}\left( P_{k} \right)}{g_{ij}}$$

Where $i,j$ and $P_{k}$ are nodes and $g_{ij}$ is the total number of shortest paths from $i$ to $j$ and $g_{ij}\left( P_{k} \right)$is the number of those paths that pass through $P_{k}$ giving a betweenness value for $P_{k}:$ $\beta\left( P_{k} \right)$ [2 3].

- 1. **Simulation model**

We present a model designed to simulate the spread of disease through a network. The probability that an individual, *i* infected an individual *j* during each time-step was given by:

$$P\left( x_{j} \right)=M_{ij}V_{j}\alpha$$

Thus, whether *i* can infect *j* depends on:

- a connection between *i* and *j* in the contact matrix *M* i.e. $M_{ij}=1;$
- on the vaccination status of j i.e. $V_{j}$;
- α is an infection constant that does not vary.

The model is implemented using R and the code is presented as a package with instructions and examples. This can be found at: <https://github.com/barryrowlingson/sirgraph>

**Available Data**

All data used during this analysis has been included in the corresponding csv files. The adjacency matrix contains the information used to create the social network (this is the raw non-dichotomised data). The file containing the influenza vaccination data uses ID codes corresponding to the codes used in the adjacency matrix, these are made up of two characters followed by a series of random numbers. The first character indicates the students year group (A = first year, B = second year, 1 = third year, 2 = fourth year and 3 = fifth year) and the second gives the students gender (X = female, Y = male).

**Data Handling**

For this analysis we dichotomised the social network at level 4 and above and assumed reciprocal ties. The following R code was used, it requires the social network data (S2 file) to be loaded into an R workspace and called “social_network_data”.

library(igraph)

m<-social_network_data

m[m=="x"]<-0

m[m==1]<-0

m[m==2]<-0

m[m==3]<-0

m<-as.matrix(m)

g <- graph.adjacency(m, weighted=NULL, mode="undirected")

g <- simplify(g)

**References**

1. Newman ME. Mixing patterns in networks. Physical Review E 2003;**67**(2):026126

2. Freeman LC. Centrality in social networks conceptual clarification. Social networks 1979;**1**(3):215-39

3. Bavelas A. A mathematical model for group structures. Human organization 1948;**7**(3):16-30
