# Supplementary material for: Seasonal Influenza Vaccination amongst Medical Students: A Social Network Analysis Based on a Cross-Sectional Study
Source: PLoS One. 2015 Oct 9;10(10):e0140085. doi: 10.1371/journal.pone.0140085 (PMC4599893; doi:10.1371/journal.pone.0140085)
Supplement: S4 File — (PDF) [file pone.0140085.s004.pdf]

Consent Form

Study Title: Medical student support structures: A social network approach

We are asking if you would like to take part in a research project. The purpose of this study is to look at the interactions between medical students and their social support structure outside their immediate peers, as well as some measures of professionalism that have been outlined in the information sheet.

Before you consent to participating in the study we ask that you read the participant information sheet and mark each box below with your initials if you agree. If you have any questions or queries before signing the consent form please speak to the principal investigator, Joseph Heath.

Please initial box after each statement

1. I confirm that I have read the information sheet and fully understand what is expected of me within this study ☐
2. I confirm that I have had the opportunity to ask any questions and to have them answered. ☐
3. I understand that my participation is voluntary and that I am free to withdraw at any time without giving any reason, without my rights as a student being affected. ☐
4. I understand that once my data have been anonymised and incorporated into themes it might not be possible for it to be withdrawn, though every attempt will be made to extract my data, up to the point of publication. ☐
5. I consent to Lancaster University keeping my data for 5 years after the study has finished. ☐
6. I consent to take part in the above study. ☐
7. I understand that any information I give will remain strictly confidential and anonymous unless it is thought that there is a risk of harm to myself or others, in which case the principal investigator may need to share this information with his research supervisor. ☐

Name of Participant___________________ **Signature**_____________________ **Date** ___________

Name of Researcher ___________________**Signature** _____________________**Date** ___________

Participant Information Sheet for Medical Students in Years 1, 2, 4, and 5

Medical student support structures: A social network approach

My name is Joseph Heath and I am conducting this research as a student in the MRes programme at Lancaster University, Lancaster, United Kingdom. My supervisor is Dr Rachel Isba

What is the study about?

The purpose of this study is to look at the interactions between medical students in the context of professionalism and follows on from studies done in the academic years 2011-12 and 2012-13. The study is looking at several things. Firstly it is looking at how well all the medical students in the school know each other and if this has changed since last time we asked. Secondly, the study also looks at two measures of professionalism – one direct (yellow cards – data for this will be obtained directly from the medical school) and one indirect (emotional intelligence). There are also a couple of questions about attitudes to having the flu vaccine.

I will only have access to the network data as the information about yellow cards, emotional intelligence, and flu vaccination is being collected for two studies being done by my supervisor, Dr Rachel Isba.

Whilst you are being asked to sign a consent form and write your name on the data collection form, I will not be allowed to see this and Dr Isba will take your name off the form and give you a code instead, before I input the data. Also, Dr Isba will be the only one allowed to look at the yellow card, emotional intelligence, and flu vaccine data, and this will also be made anonymous before being analysed. This means that nobody will be able to tell who you are or what you have put for your answers.

Why have I been approached?

You have been approached because the study is looking at medical students at Lancaster University. This study is specifically exploring Medical Student Professionalism.

Do I have to take part?

No. It's completely up to you to decide whether or not you take part. There are no negative consequence should you decide not to take part in this study.

What will I be asked to do if I take part?

If you decide you would like to take part, you will be asked to sign a consent form and then complete some data collection sheets. One of the forms will ask you about which medical students you know and how well you know them. The other forms will ask you what you think about flu vaccination and ask you to complete something called the Trait-Meta-Mood-Scale which is used as a measure of emotional intelligence.

Will my data be confidential?

The information you provide is confidential. You will be asked to write your name on the data collection sheet as it is necessary for the data analysis. However these sheets will be anonymised by Dr Isba by giving you a unique code. The data collected for this study will be stored securely in Dr Isba's office and only the researchers conducting this study will have access to the data once they have been anonymised:

- hard copies of questionnaires will be kept in a locked cabinet to which only Dr Isba has the key and, as is usual research practice, will be kept for five years before being destroyed
- computer files of anonymous data will be used on password-protected and encrypted computers only.

What will happen to the results?

The anonymous results will be summarised and reported in a thesis, may be submitted for publication in an academic or professional journal, and will be used in presentations at conferences and meetings. There will be no way of identifying you

Are there any risks?

There are no risks anticipated with participating in this study. However, if you experience any distress following participation you are encouraged to inform the researcher and seek help and advice. Some suggested resources for advice are listed at the end of this sheet.

Are there any benefits to taking part?

Although you may find participating interesting, there are currently no direct benefits in taking part.

Who has reviewed the project?

This study has been reviewed by the Faculty of Health and Medicine Research Ethics Committee, and approved by the University Research Ethics Committee at Lancaster University.

Where can I obtain further information about the study if I need it?
If you have any questions about the study, please contact the main researcher:

Joseph Heath
j.heath3@lancaster.ac.uk

Supervisor: Rachel Isba
r.isba@lancaster.ac.uk
(01524) 592450

Complaints
If you wish to make a complaint or raise concerns about any aspect of this study and do not want to speak to the researcher, you can contact:

Name of Research Director for your Division
Title: Prof. Anne Garden
Email: a.garden@lancaster.ac.uk
Tel: (01524) 593383
Lancaster Medical School
Furness Building
Lancaster University
Lancaster
LA1 4YG

If you wish to speak to someone outside of the Medical Programme, you may also contact:
Professor Paul Bates
Tel: (01524) 593718
Associate Dean for Research Email: p.bates@lancaster.ac.uk
Faculty of Health and Medicine
(Division of Biomedical and Life Sciences)
Lancaster University
Lancaster
LA1 4YD

Suggested resources for support if you experience any distress

Mind – a mental health charity
Website: www.mind.org.uk
Infoline – 0300 123 3393

Your closest local mind is at:
80-82 Devonshire Road
Chorley
Lancashire
PR7 2DR
Tel: 01257 231660
email: admin@lancashiremind.org.uk
Website: www.lancashiremind.org.uk

Samaritans – charity for emotional distress
Website: www.samaritans.org
Helpline - 08457 90 90 90
Email: jo@samaritans.org

You closest local Samaritans is at:
21 Sun Street
Lancaster
Lancashire
LA1 1EW
Tel: 01524 616666

Thank you for taking the time to read this information sheet.

Participant Information Sheet for Year 3 Medical Students

Medical student support structures: A social network approach

My name is Joseph Heath and I am conducting this research as a student in the MRes programme at Lancaster University, Lancaster, United Kingdom.

What is the study about?

The purpose of this study is to look at the interactions between medical students in the context of professionalism and follows on from studies done in the academic years 2011-12 and 2012-13. The study is looking at several things. Firstly it is looking at how well all the medical students in the school know each other and if this has changed since last time we asked. Secondly, the study also looks at two measures of professionalism – one direct (yellow cards – data for this will be obtained directly from the medical school) and one indirect (emotional intelligence). There are also a couple of questions about attitudes to having the flu vaccine.

I will only have access to the network data as the information about yellow cards, emotional intelligence, and flu vaccination is being collected for two studies being done by my supervisor, Dr Rachel Isba.

Whilst you are being asked to sign a consent form and write your name on the data collection form, I will not be allowed to see this and Dr Isba will take your name off the form and give you a code instead, before I input the data. Also, Dr Isba will be the only one allowed to look at the yellow card, emotional intelligence, and flu vaccine data, and this will also be made anonymous before being analysed. This means that nobody will be able to tell who you are or what you have put for your answers.

Why have I been approached?

You have been approached because the study is only looking at Lancaster. Also, this might be the third year that you have taken part in this study as you are part of a cohort of students (that started in 2011) that are being followed up throughout their time at medical school.

Do I have to take part?

No. It's completely up to you to decide whether or not you take part. There are no negative consequences should you decide not to take part in this study.

What will I be asked to do if I take part?

If you decide you would like to take part, you will be asked to sign a consent form and then complete some data collection sheets. One of the forms will ask you about which medical students you know and how well you know them. The other forms will ask you what you think about flu vaccination and ask you to complete something called the Trait-Meta-Mood-Scale which is used as a measure of emotional intelligence. As you are in the cohort study you are also being asked to fill in some information about who you would go to for support in various situations and a couple of questions about who you think is a good example of professional behaviour.

Will my data be confidential?

The information you provide is confidential. You will be asked to write your name on the data collection sheet as it is necessary for the data analysis. However these sheets will be anonymised by Dr Isba by giving you a unique code. The data collected for this study will be stored securely in Dr Isba's office and only the researchers conducting this study will have access to the data once they have been anonymised:

- hard copies of questionnaires will be kept in a locked cabinet to which only Dr Isba has the key and, as is usual research practice, will be kept for five years before being destroyed
- computer files of anonymous data will only be used on University, password-protected computers and will be encrypted if taken off campus

What will happen to the results?

The anonymous results will be summarised and reported in a thesis, may be submitted for publication in an academic or professional journal, and will be used in presentations at conferences and meetings. There will be no way of identifying you

Are there any risks?

There are no risks anticipated with participating in this study. However, if you experience any distress following participation you are encouraged to inform the researcher and seek help and advice. Some suggested resources for advice are listed at the end of this sheet.

Are there any benefits to taking part?

Although you may find participating interesting, there are currently no direct benefits in taking part.

Who has reviewed the project?

This study has been reviewed by the Faculty of Health and Medicine Research Ethics Committee, and approved by the University Research Ethics Committee at Lancaster University.

Where can I obtain further information about the study if I need it?
If you have any questions about the study, please contact the main researcher:

Joseph Heath
j.heath3@lancaster.ac.uk

Supervisor: Rachel Isba
r.isba@lancaster.ac.uk
(01524) 592450

Complaints
If you wish to make a complaint or raise concerns about any aspect of this study and do not want to speak to the researcher, you can contact:

Name of Research Director for your Division
Title: Prof. Anne Garden
Email: a.garden@lancaster.ac.uk
Tel: (01524) 593383
Lancaster Medical School
Furness Building
Lancaster University
Lancaster
LA1 4YG

If you wish to speak to someone outside of the Medical Programme, you may also contact:
Professor Paul Bates
Tel: (01524) 593718
Associate Dean for Research Email: p.bates@lancaster.ac.uk
Faculty of Health and Medicine
(Division of Biomedical and Life Sciences)
Lancaster University
Lancaster
LA1 4YD

Suggested resources for support if you experience any distress

Mind – a mental health charity
Website: www.mind.org.uk
Infoline – 0300 123 3393

Your closest local mind is at:
80-82 Devonshire Road
Chorley
Lancashire
PR7 2DR
Tel: 01257 231660
email: admin@lancashiremind.org.uk
Website: www.lancashiremind.org.uk

Samaritans – charity for emotional distress
Website: www.samaritans.org
Helpline - 08457 90 90 90
Email: jo@samaritans.org

You closest local Samaritans is at:
21 Sun Street
Lancaster
Lancashire
LA1 1EW
Tel: 01524 616666

Thank you for taking the time to read this information sheet.

Sample Data Collection Sheet 1 (using fictional names for illustration)

Network Analysis Sheet

This is the data collection sheet for the social network analysis. You **must** write your name in the space provided. However, your name will be coded and removed by Dr Rachel Isba, prior to data inputting and analysis. She will be the only one who sees your name, and after coding this sheet will be anonymous.

Each row contains the name of one of the medical students at the Medical School. Each column is a level of connection you have with that student.

Please place one tick only in each of the rows below

Name:

Age:

Year of study:

Sex: male / female

NAME	I do not recognise this name	I recognise this name but I would not recognise the person if I saw them	I would recognise this person if I saw them	I know this person and see or speak to them once or twice a week	I know this person and see or speak to them three or more times a week	I live with this person/have a close relationship with them/socialise with them on a regular basis
**Benjamin Gustin**	☐	☐	☐	☐	☐	☐
**Christine Hammer**	☐	☐	☐	☐	☐	☐
**Florence Brisco**	☐	☐	☐	☐	☐	☐
**Matthew Jones**	☐	☐	☐	☐	☐	☐
**Jack Brammer**	☐	☐	☐	☐	☐	☐

Sample Data Collection Sheet 2

Network Analysis Sheet

This is the data collection sheet for the emotional intelligence and flu vaccination questions. You **must** write your name in the space provided. However, your name will be coded and removed by Dr Rachel Isba, prior to data inputting and analysis. She will be the only one who sees your name, and after coding this sheet will be anonymous. These data are for Dr Isba's research and so she will be the only person using them (and also the yellow card data).

Please fill in all the questions if at all possible.

Name:

Age:

Sex: female / male

Flu vaccination questions:

Have you had your flu vaccination?

YES NO

What % of your friends do you think have had the flu vaccination?

%

What % of Lancaster medical students do you think have had the flu vaccination?

%

What % of doctors do you think have had the flu vaccination?

%
